# Characteristics of Cancer-Associated Thrombosis Based on Changes in Clinically Measurable Coagulation Factors and Coagulation/Fibrinolysis Markers in Patients with Cancer: A Multicenter Cross-Sectional Study

**DOI:** 10.3400/avd.oa.26-00081

**Published:** 2026-09-12

**Authors:** Makoto Hiraide, Chikao Yasuda, Ryuji Kawaguchi, Masakatsu Paku, Nariaki Fukuchi, Yoshihiro Matsumoto, Toshiki Takemoto, Kohei Tatsumi

**Affiliations:** 1 Department of Clinical Assessment, Tokyo University of Pharmacy and Life Sciences, Tokyo, Japan; 2 Division of Vascular Surgery, Attra Shinmachi Clinic, Hirakata, Osaka, Japan; 3 Department of Obstetrics and Gynecology, Nara Medical University, Kashihara, Nara, Japan; 4 Department of Gastroenterological Surgery, Osaka Keisatsu Hospital, Osaka, Osaka, Japan; 5 Department of Surgery, Japan Community Health Care Organization, Hoshigaoka Medical Center, Hirakata, Osaka, Japan; 6 Department of Urology, Japan Community Health Care Organization, Hoshigaoka Medical Center, Hirakata, Osaka, Japan; 7 Division of Thoracic Surgery, Department of Surgery, Kindai University Faculty of Medicine, Sakai, Osaka, Japan; 8 Advanced Medical Science of Thrombosis and Hemostasis, Nara Medical University, Kashihara, Nara, Japan

**Keywords:** biomarker, cancer-associated thrombosis, coagulation, fibrinolysis, venous thromboembolism

## Abstract

**Objectives:**

This exploratory, cross-sectional study aimed to examine the association between coagulation factor activity and coagulation/fibrinolysis markers and the presence of cancer-associated thrombosis (CAT).

**Methods:**

Patients diagnosed with cancer complicated by thrombosis and those without thrombosis between November 1, 2020, and December 31, 2022 were included. The clinical characteristics and values of each coagulation factor and coagulation/fibrinolysis marker in the CAT and non-CAT groups were compared.

**Results:**

Of the 117 patients analyzed, 22 (18.8%) had CAT and 95 (81.2%) did not have CAT. Fibrinogen/fibrin degradation product (FDP), D-dimer, alpha2-plasmin inhibitor–plasmin complex (PIC), fibrin monomer complex (FMC), thrombin–antithrombin complex (TAT), and soluble fibrin (SF) levels were significantly higher in the CAT group than that in the non-CAT group. Receiver operating characteristic curve analysis was performed, and the proportions of patients with CAT were 7.9% when the number of factors with a value higher than the cutoff was 0 among the 6 factors of FDP, D-dimer, PIC, FMC, TAT, and SF levels; 17.1% for factors 1–3 and 31.6% for factors 4–6.

**Conclusions:**

When hypercoagulability and hyperfibrinolysis coexist in patients with cancer, CAT is likely to be present, and it is necessary to confirm the presence of CAT at an early stage.

## Introduction

Although new anticancer drugs have emerged in recent years, cancer therapy-related cardiovascular toxicity (CTR-CVT) is prevalent, and its early detection and response are urgent issues.^1–3)^ CTR-CVT includes cancer therapy-related cardiac dysfunction, abnormal blood pressure, abnormal electrocardiogram findings/arrhythmia (bradycardia, QT prolongation), ischemic heart disease, thromboembolism, and myocarditis. Cancer-associated thrombosis (CAT) is one of the major causes of death among cancer survivors.^4)^

CAT is not only a general term for thrombosis occurring in patients with cancer, including venous thromboembolism (VTE) such as deep vein thrombosis (DVT) and pulmonary embolism (PE), but also arterial thromboembolism such as cerebral infarction, myocardial infarction, and peripheral arterial disease. CAT can also involve thrombus formation at various sites, including the portal and pelvic veins, as well as the ports and catheter implantation sites. Other associated conditions include nonbacterial thromboendocarditis and disseminated intravascular coagulation (DIC) syndrome.^5)^

Cancer-associated VTE (CAVT), the most frequent form of CAT, is found in 10%–20% of hospitalized patients with cancer.^6)^ Reports from the early 2000s indicated that the risk of developing VTE in patients with cancer was 4.1–6.5 times higher than that in patients without cancer and that the incidence was up to 47 times higher in patients with cancer receiving anticancer drug therapy than in those not receiving therapy.^7)^ In addition, Mulder et al. reported that the incidence of VTE in patients with cancer was 9.1 times higher than that in patients without cancer and that the incidence of VTE in patients with cancer increased 3-fold during the 7-year period from 2011 to 2017 compared with the 7-year period from 1997 to 2003 and increased 6-fold in patients receiving chemotherapy.^8)^ The importance of early prediction of CAVT onset has recently been emphasized. Furthermore, in a registry study of cancer-related thrombosis in Japanese subjects (COMMAND Cancer VTE Registry-2), patients with active cancer were found in 29% of the registered acute symptomatic VTE cases, and the increase in this incidence is an issue in Japan, as it is in Europe and the United States.^9)^

Coagulation/fibrinolytic function may have an important role in the mechanism linking cancer and thrombosis.^10,11)^ The main factors that cause abnormal coagulation and fibrinolysis in cancer cells are (1) thrombin overexpression by endogenous or exogenous stimuli in the coagulation cascade and activation of factor XI by the produced thrombin^12)^; (2) activation of coagulation factors via factor Xa,^13)^ which is induced by exogenous coagulation reactions owing to the tissue factor expression; (3) formation of arterial thrombus by promoting platelet aggregation; (4) decreased production of antithrombotic substances, such as thrombomodulin (TM) and antithrombin (AT), by producing various inflammatory cytokines (interleukin [IL]-1, IL-6, and tumor necrosis factor [TNF]-α)^14)^; and (5) decreased fibrinolytic function due to the expression of plasminogen activator inhibitor-1 (PAI-1), among many other possible mechanisms.^15,16)^ However, no conclusions have been drawn regarding the relevance of each biomarker in CAT or the differences based on cancer type.

Recently, automated analysis of coagulation and fibrinolysis markers with reagents has enabled the analysis of many hypercoagulability markers, such as the thrombin–AT complex (TAT), fibrin monomer complex (FMC), and soluble fibrin (SF), and hyperfibrinolysis markers, such as D-dimer, alpha2-plasmin inhibitor–plasmin complex (PIC), and fibrinogen/fibrin degradation product (FDP). These automated analyzers are capable of measuring multiple items with only a small sample volume; moreover, there is the potential to compare the accuracy and relevance of CAT with multiple biomarkers that were previously unexamined.

We aimed to investigate the relationship between coagulation factor activity and coagulation/fibrinolysis markers, which can be measured using automated analyzers, and the presence of CAT, to assess which factors have a strong influence on coagulation/fibrinolysis dynamics in patients with cancer and how they are associated with CAT.

## Materials and Methods

### Study design

This multicenter cross-sectional study was conducted in accordance with the ethical principles set forth in the Declaration of Helsinki and the relevant Japanese regulations regarding ethics and data protection. This study was approved by the ethics committee of each participating institution before implementation, and written informed consent was obtained from all patients before participation (ethics review approval number: 2033). In this cross-sectional design, patients with cancer were classified by the presence or absence of CAT, and each patient was assessed at a single time point.

### Patients

This study included patients diagnosed with cancer complicated by thrombosis and those without thrombosis between November 1, 2020, and December 31, 2022. The inclusion criteria were as follows: (1) age ≥18 years, (2) diagnosis of a solid tumor, (3) active cancer status (defined as receiving radiation or systemic therapy, or having been diagnosed with cancer within 2 years or at an advanced stage), and (4) Performance Status (PS) of 0–2. The following patients were excluded from this study: (1) patients who were expected to live for <3 months, (2) pregnant or lactating patients, and (3) patients who were deemed inappropriate by the physician in charge. Patients were enrolled at the outpatient clinics of the participating institutions (not a commercial registry); eligible patients were enrolled consecutively where feasible.

### Data collection

The following clinical data were collected at the time of enrollment in this study. Patient characteristics included sex, age, height, weight, smoking status, comorbidities, PS, type of malignancy, stage, presence of thrombus, and, if a thrombus was present, type of thrombosis and anticoagulation therapy, as well as laboratory values (serum creatinine, creatinine clearance, total bilirubin, white blood cell, hemoglobin, platelet, C-reactive protein [CRP]). Fibrinogen, II, V, VII, VIII, IX, X, XI, XII, and XIII factor activity as coagulation factors, prothrombin time-international normalized ratio (PT-INR), activated partial thromboplastin time (APTT), FDP, D-dimer, PIC, FMC, TAT, SF, alpha2-plasmin inhibitor (alpha2-PI), PAI-1, TM, plasminogen (PLG), AT, and anti-Xa activity were collected as biomarkers of the coagulation/fibrinolytic system. All of these parameters were measured for this study, and the results were also used in the patients' clinical management. The routine coagulation tests (PT-INR, APTT, FDP, and D-dimer) and general laboratory values are generally available in clinical practice, whereas the coagulation factor activities and the other specialized markers are not routinely measured.

### Outcomes

In this study, the coagulation factor and coagulation/fibrinolysis marker values in eligible patients with active cancer were used as outcomes. The patients were classified into 2 groups, CAT and non-CAT, based on the presence or absence of CAT. The clinical characteristics and values of each coagulation factor and coagulation/fibrinolysis marker in the 2 groups were compared. In accordance with the pre-specified study protocol, CAT was defined as both venous and arterial thromboembolism, confirmed by appropriate imaging, and included both symptomatic and asymptomatic events; arterial events were included when clinically adjudicated as cancer-associated. The asymptomatic events were detected incidentally on imaging performed for cancer staging or management, which patients in both groups underwent to a comparable extent, and no dedicated systematic screening was applied. Eligible thrombi included lower or upper extremity (jugular, sternal, subclavian, axillary, upper arm), DVT, PE, splenic (hepatic, portal, splenic, mesenteric, renal, gonadal), or cerebral venous thrombosis, as well as arterial thromboembolism (e.g., cerebral infarction, peripheral arterial thrombosis, and aortic thrombosis), as identified by appropriate imaging modalities. In the CAT group, blood samples were obtained at the time thrombosis was present, whereas in the non-CAT group they were obtained at the initiation of cancer treatment. In addition, data from patients not receiving anticoagulants were analyzed to determine their effects on coagulation factors and coagulation/fibrinolysis markers. The data for all 134 patients, including those on anticoagulants, are presented in **Supplementary Tables 1–3**.

### Measurement

Three 5-mL whole blood samples were collected using 3.2% citrate plasma. The samples were centrifuged twice at 2000 × *g* for 15 min and examined after cryopreservation. Coagulation factor activity was tested using standard protocols with fully automated hemostasis analyzers (CN-3000/CN-6000; Sysmex, Hyogo, Japan). Coagulation factor VIII and IX activities were measured using the synthetic substrate method, whereas other coagulation factor activities were measured using the 1-stage coagulation method. To test coagulation and fibrinolysis markers, PT-INR, APTT, FMC, PLG, and AT were assessed using a CN-3000/CN-6000 (Sysmex). FDP, D-dimer, and anti-Xa activity were analyzed using ACL TOP 550 CTS (Werfen, Barcelona, Spain), and PIC, TAT, SF, α2-PI, PAI-1, and TM were analyzed using STACIA (PHC, Tokyo, Japan). All reagents and calibrators were matching reagents obtained from the original manufacturer and used according to the manufacturer’s instructions.

### Statistical analyses

Descriptive statistics (frequencies, percentages, means, and standard deviations) were used to summarize the data. After confirming a normal distribution, we compared the diseases, characteristics, and biomarkers with and without CAT using the t-test, Pearson’s chi-squared test, and Fisher’s exact test. For each biomarker that showed differences in the 2-group comparison, the cutoff values were estimated using receiver operating characteristic (ROC) curve analysis. The significance level was set at p <0.05, and statistical analyses were performed using R version 4.4.1. This study was exploratory and hypothesis-generating; no adjustment for multiple comparisons was made, and the ROC-derived cutoff values were derived and evaluated in the same sample and should be regarded as exploratory and unvalidated.

## Results

### Study population

**Fig. 1:**
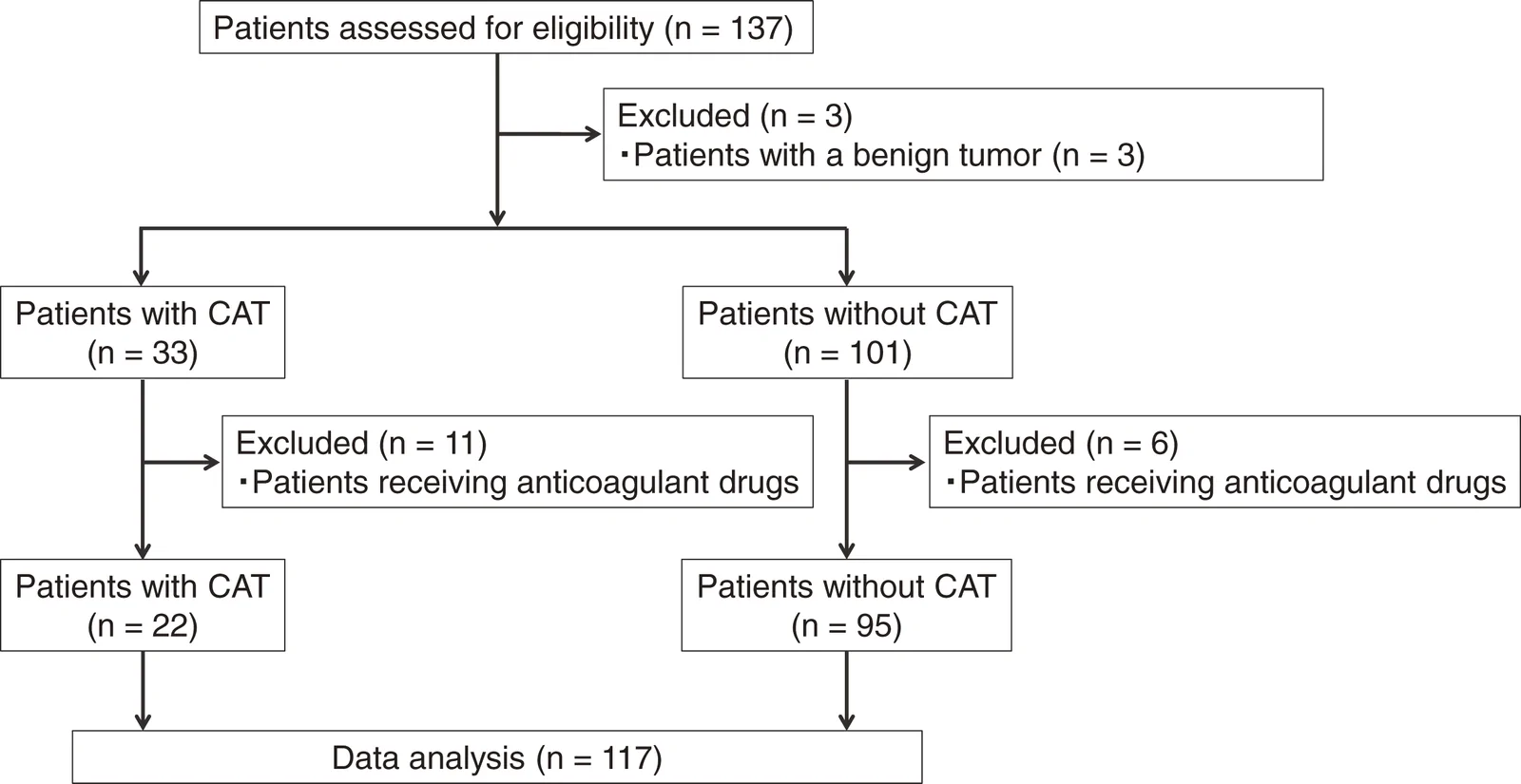
Selection of the study cohort.

In total, 137 patients were enrolled in this study. Three patients with a diagnosis of benign tumors and 17 patients on anticoagulants were excluded; finally, 117 patients were analyzed (**Fig. 1**). Of these, 22 (18.8%) were CAT cases, and 95 (81.2%) were non-CAT cases. The patient backgrounds in each group are shown in **Table 1**. The mean age of all patients was 71.5 ± 9.7 years, and 65 (55.6%) were men. Among the eligible patients, the most frequent tumors were gastrointestinal and genitourinary cancers in 65 (55.6%) and 24 (20.5%) patients, respectively, and distant metastasis was observed in 32 (27.4%) patients. There were significantly more patients with PS 2 in the CAT group than that in the non-CAT group (18.2% vs. 3.2%, p = 0.024) and a significantly lower rate of gastrointestinal cancer in the CAT group than that in the non-CAT group (31.8% vs. 61.1%, p = 0.017). In laboratory values, CRP was significantly higher in the CAT group than that in the non-CAT group (2.41 vs. 0.84, p = 0.043). Among the 22 CAT cases, 10 were symptomatic and 12 were asymptomatic, and 17 were venous and 5 were arterial (3 cerebral infarctions, 1 peripheral arterial thrombosis, and 1 abdominal aortic thrombosis), all clinically adjudicated as cancer-associated. The distribution of thrombosis types and the prevalence of CAT by cancer type are presented in **Supplementary Table 4**.

**Table 1:** Characteristics of the study population

	All	CAT	Non-CAT	p-Value
	n = 117	n = 22	n = 95	
Sex^a^				
Male	65 (55.6)	9 (40.9)	56 (58.9)	0.155
Female	52 (44.4)	13 (59.1)	39 (41.1)	0.155
Age (years)^b^	71.5 ± 9.7	74.1 ± 9.5	70.9 ± 9.7	0.172
Weight (kg)^b^	59.5 ± 11.2	60.0 ± 10.0	59.4 ± 11.4	0.868
BMI (kg/m^2^)^b^	22.8 ± 4.3	23.2 ± 3.8	22.7 ± 4.3	0.594
Smoking^a^				
Non	65 (55.6)	13 (59.1)	52 (54.7)	0.814
Former	29 (24.8)	4 (18.2)	24 (25.3)	1.000
Current	23 (19.7)	5 (22.7)	19 (20.0)	1.000
Diabetes mellitus^a^	28 (23.9)	7 (31.8)	21 (22.1)	0.406
PS^a^				
0	96 (82.1)	16 (72.7)	80 (84.2)	0.209
1	14 (12.0)	2 (9.1)	12 (12.6)	1.000
2	7 (6.0)	4 (18.2)	3 (3.2)	0.024
Type of cancer^a^				
Lung	10 (8.5)	2 (9.1)	8 (8.4)	1.000
Gastrointestinal	65 (55.6)	7 (31.8)	58 (61.1)	0.017
Genitourinary	24 (20.5)	8 (36.4)	16 (16.8)	0.091
Gynecological	16 (13.7)	5 (4.3)	11 (11.6)	0.179
Others	2 (1.7)	0 (0.0)	2 (2.1)	1.000
Cancer stage at diagnosis^a^				
Local	38 (32.5)	6 (27.3)	32 (33.7)	0.800
Recurrent locally advanced	47 (40.2)	6 (27.3)	41 (43.2)	0.242
Metastatic	32 (27.4)	10 (45.5)	22 (23.2)	0.065
Laboratory values^b^				
SCr (mg/dL)	0.89 ± 0.47	0.94 ± 0.89	0.87 ± 0.30	0.710
CCr (mL/min)	65.7 ± 22.4	65.7 ± 25.8	65.7 ± 21.6	0.943
T-Bil (mg/dL)	0.61 ± 0.34	0.63 ± 0.39	0.61 ± 0.33	0.775
WBC (/μL)	6284 ± 2421	6659 ± 2490	6197 ± 2396	0.445
Hb (g/dL)	12.3 ± 2.1	12.2 ± 1.9	12.3 ± 2.1	0.757
PLT (×10^3^/μL)	24.8 ± 9.9	25.9 ± 12.3	24.6 ± 9.2	0.633
CRP (mg/dL)	1.14 ± 2.18	2.41 ± 3.29	0.84 ± 1.70	0.043

^a^n (%).

^b^Mean ± standard deviation.

BMI, body mass index; PS, performance status; SCr, serum creatinine; CCr, creatinine clearance; WBC, white blood cell; Hb, hemoglobin; PLT, platelet; CRP, C-reactive protein; CAT, cancer-associated thrombosis

### Coagulation factor activity

The activity of each coagulation factor in the CAT and non-CAT groups is presented in **Table 2**. Both groups had fibrinogen levels at the upper end of the normal range (200–400 mg/dL) and factor VIII levels above the normal range (60%–140%). A significantly lower factor XII level was also found in the CAT group than that in the non-CAT group (60.9% vs. 75.1%, p = 0.035).

**Table 2:** Comparison of coagulation factor activity in patients with and without cancer-associated thrombosis

Coagulation factor activity^a^	Reagent manufacturer	Normal range	All	CAT	Non-CAT	p-Value
			n = 117	n = 22	n = 95	
Fibrinogen (mg/dL)	Sysmex	200–400	396.1 ± 136.2	404.9 ± 141.6	394.0 ± 134.8	0.738
II (%)	Sysmex	60–140	90.3 ± 16.9	87.8 ± 12.9	90.9 ± 17.6	0.440
V (%)	Sysmex	60–140	125.6 ± 22.1	132.5 ± 16.5	124.1 ± 22.9	0.110
VII (%)	Sysmex	60–140	99.8 ± 24.7	102.4 ± 22.7	99.2 ± 25.1	0.590
VIII (%)	Sysmex	60–140	176.2 ± 56.0	180.4 ± 44.6	175.2 ± 58.3	0.697
IX (%)	Sysmex	60–140	107.1 ± 23.3	111.3 ± 17.1	106.1 ± 24.4	0.349
X (%)	Sysmex	60–140	83.6 ± 18.6	78.4 ± 17.4	84.7 ± 18.7	0.154
XI (%)	Sysmex	60–140	79.9 ± 20.5	78.9 ± 16.1	80.1 ± 21.4	0.811
XII (%)	Sysmex	60–140	72.4 ± 28.5	60.9 ± 23.8	75.1 ± 28.8	0.035
XIII (%)	Sysmex	70–140	83.5 ± 23.2	76.4 ± 20.9	85.1 ± 23.4	0.114

^a^Mean ± standard deviation.

CAT, cancer-associated thrombosis

### Coagulation and fibrinolysis markers

The values of each coagulation/fibrinolysis marker in the CAT and non-CAT groups are presented in **Table 3**. The FDP, D-dimer, PIC, FMC, TAT, and SF levels were above the reference values in both groups. FDP (19.2 vs. 3.6 μg/mL, p = 0.001), D-dimer (7.44 vs. 1.79 μg/mL FEU, p <0.001), PIC (2.63 vs. 1.16 μg/mL, p = 0.017), FMC (89.9 vs. 11.7 μg/mL, p = 0.035), TAT (8.62 vs. 2.41 ng/mL, p = 0.002), and SF (21.7 vs. 7.7 μg/mL, p = 0.016) levels were significantly higher in the CAT group than that in the non-CAT group.

**Table 3:** Comparison of coagulation and fibrinolysis markers in patients with and without cancer-associated thrombosis

Marker	Reagent manufacturer	Normal range	All	CAT	Non-CAT	p-Value
			n = 117	n = 22	n = 95	
General coagulation tests^a^						
PT-INR	Sysmex	0.70–1.30	0.97 ± 0.09	0.97 ± 0.08	0.98 ± 0.10	0.637
APTT (seconds)	Sysmex	24–34	29.8 ± 17.5	28.0 ± 2.5	30.3 ± 19.4	0.581
Coagulation and fibrinolysis markers^a^						
FDP (μg/mL)	Werfen	0–5.2	6.5 ± 20.8	19.2 ± 43.8	3.6 ± 6.5	0.001
D-dimer (μg/mL FEU)	Werfen	0–0.6	2.85 ± 7.29	7.44 ± 14.63	1.79 ± 3.14	<0.001
PIC (μg/mL)	PHC	0–0.8	1.44 ± 2.60	2.63 ± 5.60	1.16 ± 0.80	0.017
FMC (μg/mL)	Sysmex	≤6.1	26.4 ± 156.2	89.9 ± 343.1	11.7 ± 40.7	0.035
TAT (ng/mL)	PHC	0–3	3.58 ± 8.48	8.62 ± 16.06	2.41 ± 4.65	0.002
SF (μg/mL)	PHC	0–7	10.4 ± 24.6	21.7 ± 42.6	7.7 ± 16.9	0.016
α2-PI (%)	PHC	77–120	85.1 ± 13.1	85.6 ± 12.1	85.0 ± 13.3	0.819
PAI-1 (ng/mL)	PHC	0–50	20.0 ± 10.8	24.0 ± 9.4	19.1 ± 10.9	0.059
TM (U/mL)	PHC	8.7–22.7	19.9 ± 9.5	21.1 ± 11.5	19.7 ± 9.0	0.530
PLG (%)	Sysmex	80–130	92.6 ± 19.4	88.7 ± 19.3	93.5 ± 19.3	0.292
AT (%)	Sysmex	80–130	95.3 ± 15.0	91.5 ± 12.4	96.2 ± 15.4	0.182
Anti-Xa activity (IU/mL)	Werfen	–	0.02 ± 0.08	0.01 ± 0.01	0.02 ± 0.09	0.557

^a^Mean ± standard deviation.

CAT, cancer-associated thrombosis; PT-INR, prothrombin time-international normalized ratio; APTT, activated partial thromboplastin time; FDP, fibrinogen/fibrin degradation products; PIC, alpha2-plasmin inhibitor–plasmin complex; FMC, fibrin monomer complex; TAT, thrombin–antithrombin complex; SF, soluble fibrin; α2-PI, alpha2-plasmin inhibitor; PAI-1, plasminogen activator inhibitor-1; TM, thrombomodulin; PLG, plasminogen; AT, antithrombin

The cutoff values for FDP, D-dimer, PIC, FMC, TAT, and SF using ROC curve analysis were 2.15 μg/mL (area under the curve [AUC], 0.73), 1.74 μg/mL FEU (AUC, 0.72), 3.70 μg/mL (AUC, 0.65), 1.15 μg/mL (AUC, 0.64), 3.15 ng/mL (AUC, 0.61), and 2.62 μg/mL (AUC, 0.66), respectively. For each of these 6 factors, the number of factors with values above the cutoff value in each patient was calculated, and the proportions of patients with CAT according to the number of factors (0, 1–3, 4–6) are shown in **Table 4**. The proportions of patients with CAT were 3/38 (7.9%) when the number of factors with values above the cutoff was 0, 7/41 (17.1%) when the number of factors was 1–3, and 12/38 (31.6%) when the number of factors was 4–6.

**Table 4:** Relationship between the number of coagulation/fibrinolysis marker factors and the incidence of cancer-associated thrombosis

Factors	CAT, n	Total, n	Incidence of CAT (%)
0	3	38	7.9
1–3	7	41	17.1
4–6	12	38	31.6

For each of the 6 factors, FDP, D-dimer, PIC, FMC, TAT, and SF, we counted the number of factors showing a cutoff value above that indicated in the receiver operating characteristic curve analysis for each patient.

CAT, cancer-associated thrombosis; FMC, fibrin monomer complex; FDP, fibrin degradation product; PIC, alpha2-plasmin inhibitor–plasmin complex; TAT, thrombin–antithrombin complex; SF, soluble fibrin

## Discussion

This study aimed to investigate how coagulation and fibrinolysis functions are balanced in patients with cancer by simultaneously assessing multiple coagulation factor activities and coagulation and fibrinolysis markers that can be measured using automated analyzers and to clarify the relationship between CAT development and each biomarker.

In healthy living organisms, blood is balanced to prevent clotting by inhibiting coagulation via vascular endothelial cells and regulating the fibrinolytic system. However, the risk of developing CAT is increased in patients with cancer owing to patient-related factors, such as advanced age and prolonged bed rest; cancer-related factors owing to the type and stage of cancer; and treatment-related factors owing to surgery and radiation, hormone, and cancer drug therapies.^8)^ In this study, fewer patients with gastrointestinal cancer had CAT when comparing patient backgrounds between the CAT and non-CAT groups. Among gastrointestinal cancers, pancreatic cancer, in particular, has a high risk of developing thrombus.^17–19)^ In this study, the low proportion of patients with pancreatic cancer (5/65 [7.7%]) was considered to be one reason for the significantly lower number of CAT cases in the gastrointestinal cancer group (p = 0.017). In addition, the CRP levels were significantly higher in the CAT group than that in the non-CAT group (p = 0.043). Regarding CRP, inflammatory cytokines, such as IL-6 and TNF-α, activate the vascular endothelium and promote thrombus formation in patients with cancer.^20)^ The same mechanism may have contributed to the high CRP levels observed in the CAT group in this study.

Our study measured multiple coagulation factor activities and revealed significantly lower factor XII activity in the CAT group than that in the non-CAT group (60.9% vs. 75.1%, p = 0.035). Factor XII increases the production of factor XIa owing to thrombin overproduction.^12)^ These results suggest that factor XII activity may have been low due to the reduced supply of factor-activated XII (XIIa). In both groups, the mean fibrinogen level was at the upper limit of normal, and the mean factor VIII activity was largely above the upper limit of normal. As for fibrinogen, it increases with the degree of cancer progression,^21)^ and the results were similar to those previously reported. Furthermore, fibrinogen is a useful indicator of hypercoagulability and may have influenced our data.^22)^ An increase in von Willebrand factor (vWF) antigen levels may be involved in factor VIII activity.^23)^ As this study did not present data on vWF antigen levels and the mechanism was not clarified; we believe that further data accumulation is required.

Regarding the coagulation and fibrinolysis markers, FDP, D-dimer, PIC, FMC, TAT, and SF levels were higher than normal in both groups. Furthermore, FDP, D-dimer, PIC, FMC, TAT, and SF levels were significantly higher in the CAT group than that in the non-CAT group. Among the 6 markers that were significantly elevated in the CAT group, FDP, D-dimer, and PIC were evaluated as markers of fibrinolysis, whereas FMC, TAT, and SF were evaluated as markers of hypercoagulability, each of which has been used in clinical practice. Although the strength of the association between each marker and CAT development was not clear in this study, it is necessary to consider the possibility that the higher values of the markers elevated in this study may increase the likelihood of thrombosis and DIC.

We also performed ROC curve analysis of the 6 coagulation/fibrinolysis markers that were elevated in the CAT group and calculated their respective cutoff values. The results of the ROC analysis suggest that FDP, D-dimer, PIC, FMC, TAT, and SF have diagnostic value for thrombosis in patients with cancer. For these 6 markers, the proportion of CAT cases increased as the number of factors (0, 1–3, 4–6) exceeding cutoff values increased. If 4 or more factors exceeded their cutoff values, the case was likely to indicate simultaneous hypercoagulation and hyperfibrinolysis. Therefore, if both coagulation and fibrinolysis markers were elevated, it would be more likely that the patient had CAT (i.e., that CAT was present). From a practical standpoint, although D-dimer showed high sensitivity, FDP also exceeded an area under the ROC curve of 0.70 and may serve as an alternative when D-dimer measurement is unavailable. Conversely, the hypercoagulability markers FMC, TAT, and SF cannot be measured at many institutions, so combining markers of hyperfibrinolysis and hypercoagulability may aid the early diagnosis of CAT.

This study has some limitations. First, although this study enrolled patients from multiple centers, the sample size made it challenging to adequately assess the differences in biomarker utility for each tumor type. Therefore, the usefulness of the factors that were elevated in the CAT group should be tested in further studies with a larger number of patients. A second limitation was the need to collect blood samples from multiple sites owing to the multicenter nature of this study. Although the method of biomarker measurement was defined in advance, there were concerns that variations in the time from blood collection to plasma separation, as well as differences in the timing of blood collection across facilities, could have influenced the results. In addition, it was difficult to measure the active forms of the coagulation factors using the automated analyzers used in this study, and we only measured the activity values of the coagulation factors. Future studies are required to elucidate the regulatory mechanisms underlying active coagulation factors. Finally, with only 22 CAT events, the data were insufficient for reliable multivariable adjustment, so the between-group differences are unadjusted and should be regarded as exploratory. Undetected asymptomatic thrombosis in the non-CAT group and the inclusion of a small number of arterial events with distinct pathophysiology may also have influenced the results, and these findings require confirmation in larger studies.

## Conclusion

In this cross-sectional study of patients with cancer, the concurrent elevation of hypercoagulability markers (FMC, TAT, and SF) and hyperfibrinolysis markers (FDP, D-dimer, and PIC) was associated with the presence of CAT, with the proportion of patients with CAT increasing as the number of markers exceeding their cutoff values increased. Combined measurement of coagulation and fibrinolysis markers may aid the early diagnosis of CAT in patients with cancer. Given the small sample size, the differing timing of blood sampling between the groups, and the possible misclassification of asymptomatic CAT, these findings should be regarded as exploratory and require confirmation in larger studies.

## Supplementary Materials

Supplementary Table 1 Characteristics of the study population.

Supplementary Table 2 Comparison of coagulation factor activity in patients with and without cancer-associated thrombosis.

Supplementary Table 3 Comparison of coagulation markers in patients with and without cancer-associated thrombosis.

Supplementary Table 4 Prevalence of cancer-associated thrombosis (CAT) by cancer type, with venous and arterial breakdown.
