# Supplementary material for: Characteristics of Cancer-Associated Thrombosis Based on Changes in Clinically Measurable Coagulation Factors and Coagulation/Fibrinolysis Markers in Patients with Cancer: A Multicenter Cross-Sectional Study
Source: Ann Vasc Dis. 2026 Sep 12;19(1):26-00081. doi: 10.3400/avd.oa.26-00081 (PMC13581452; doi:10.3400/avd.oa.26-00081)
Supplement: Supplementary file 1 [file avd-19-1-26-00081-s001.pdf]

Supplementary Table 1 Characteristics of the study population

	All n = 134	CAT n = 33	Non-CAT n = 101	p-value
Sex[a]				
Male	74 (55.2)	15 (45.5)	59 (58.4)	0.229
Female	60 (44.8)	18 (54.5)	42 (41.6)	0.229
Age (years)[b]	72.2 ± 9.6	74.0 ± 8.8	71.6 ± 9.8	0.210
Weight (kg)[b]	59.6 ± 11.5	60.2 ± 10.9	59.4 ± 11.7	0.819
BMI (kg/m^2)[b]	22.9 ± 4.7	22.9 ± 5.5	22.9 ± 4.4	0.954
Smoking[a]				
Non	77 (57.5)	20 (60.6)	57 (56.4)	0.691
Former	31 (23.1)	6 (18.2)	25 (24.8)	0.487
Current	26 (19.4)	7 (21.2)	19 (18.8)	0.802
Diabetes mellitus[a]	33 (24.6)	9 (27.3)	24 (23.8)	0.816
PS[a]				
0	110 (82.1)	24 (72.7)	86 (85.1)	0.110
1	17 (12.7)	5 (15.2)	12 (11.9)	0.543
2	7 (5.2)	4 (12.1)	3 (3.0)	0.064
Type of cancer[a]				
Lung	12 (9.0)	4 (12.1)	8 (7.9)	0.489
Gastrointestinal	74 (55.2)	12 (36.4)	62 (61.4)	0.016
Genitourinary	28 (20.9)	11 (33.3)	17 (16.8)	0.056
Gynecological	18 (13.4)	6 (18.2)	12 (11.9)	0.383
Others	2 (1.5)	0 (0.0)	2 (2.0)	1.000
Cancer stage at diagnosis[a]				
Local	38 (28.4)	7 (21.2)	31 (30.7)	0.529
Recurrent locally advanced	57 (42.5)	13 (39.4)	44 (43.6)	0.691
Metastatic	39 (29.1)	13 (39.4)	26 (25.7)	0.185
Anticoagulant therapy[a]	17 (12.7)	11 (33.3)	6 (5.9)	< 0.001
Laboratory values[b]				
SCr (mg/dL)	0.90 ± 0.46	0.90 ± 0.75	0.90 ± 0.32	0.987
CCr (mL/min)	64.3 ± 23.2	66.4 ± 24.2	63.6 ± 22.9	0.567
T-Bil (mg/dL)	0.64 ± 0.46	0.73 ± 0.73	0.61 ± 0.33	0.393
WBC (/μL)	6399 ± 2543	7179 ± 2858	6144 ± 2376	0.043
Hb (g/dL)	12.2 ± 2.1	11.8 ± 1.9	12.3 ± 2.1	0.240
PLT ($\times 10^3/\mu L$)	25.4 ± 10.7	28.7 ± 14.1	24.3 ± 9.1	0.039
CRP (mg/dL)	1.24 ± 2.49	2.53 ± 3.79	0.81 ± 1.65	< 0.001

[a]n (%)

[b]Mean ± standard deviation

BMI, body mass index; PS, Performance Status; SCr, serum creatinine; CCr, creatinine clearance; WBC, white blood cell; Hb, hemoglobin; PLT, platelet; CRP, C-reactive protein; CAT, cancer-associated thrombosis

Supplementary Table 2 Comparison of coagulation factor activity in patients with and without cancer-associated thrombosis

Coagulation factor activity[a]	Reagent manufactures	Normal range	All n = 134	CAT n = 33	Non-CAT n = 101	p-value
Fibrinogen (mg/dL)	Sysmex	200–400	394.7 ± 132.3	406.1 ± 129.4	391.0 ± 133.0	0.572
II (%)	Sysmex	60–140	89.6 ± 16.2	86.3 ± 12.5	90.7 ± 17.2	0.175
V (%)	Sysmex	60–140	124.6 ± 21.7	128.8 ± 17.9	123.5 ± 22.8	0.335
VII (%)	Sysmex	60–140	98.6 ± 25.6	94.7 ± 27.1	99.9 ± 24.9	0.312
VIII (%)	Sysmex	60–140	177.7 ± 58.0	187.4 ± 59.7	174.5 ± 57.1	0.269
IX (%)	Sysmex	60–140	106.0 ± 22.7	108.0 ± 16.2	105.3 ± 24.4	0.563
X (%)	Sysmex	60–140	81.6 ± 18.5	74.5 ± 16.9	83.9 ± 18.5	0.012
XI (%)	Sysmex	60–140	79.6 ± 20.6	79.0 ± 17.9	79.8 ± 21.4	0.860
XII (%)	Sysmex	60–140	71.6 ± 28.5	64.0 ± 27.3	74.1 ± 28.5	0.077
XIII (%)	Sysmex	70–140	83.3 ± 24.4	76.1 ± 26.4	85.7 ± 23.3	0.051

[a]Mean ± standard deviation

CAT, cancer-associated thrombosis

Supplementary Table 3 Comparison of coagulation marker in patients with and without cancer-associated thrombosis

Marker	Reagent manufacturer	Normal range	All n = 134	CAT n = 33	Non-CAT n = 101	p-value
General coagulation test[a]						
PT-INR	Sysmex	0.70–1.30	0.99 ± 0.12	1.03 ± 0.16	0.98 ± 0.09	0.034
APTT (seconds)	Sysmex	24–34	30.7 ± 17.4	31.9 ± 11.6	30.3 ± 18.9	0.643
Coagulation fibrinolysis marker[a]						
FDP (μg/mL)	Werfen	0–5.2	6.9 ± 20.0	17.1 ± 36.9	3.5 ± 6.3	< 0.001
D-dimer (μg/mL FEU)	Werfen	0–0.6	3.04 ± 7.19	7.05 ± 12.63	1.73 ± 3.06	< 0.001
PIC (μg/mL)	PHC	0–0.8	1.43 ± 2.44	2.30 ± 4.62	1.15 ± 0.78	0.018
FMC (μg/mL)	Sysmex	≤ 6.1	24.8 ± 146.6	66.2 ± 283.1	11.2 ± 39.5	0.062
TAT (ng/mL)	PHC	0–3	3.57 ± 8.05	7.22 ± 13.53	2.38 ± 4.53	0.003
SF (μg/mL)	PHC	0–7	10.8 ± 26.8	21.2 ± 44.2	7.4 ± 16.5	0.011
α2−PI (%)	PHC	77–120	85.1 ± 12.6	86.2 ± 11.5	84.7 ± 13.0	0.554
PAI-1 (ng/mL)	PHC	0–50	21.7 ± 15.0	29.4 ± 21.9	19.2 ± 10.8	< 0.001
TM (U/mL)	PHC	8.7–22.7	20.1 ± 9.2	20.9 ± 9.8	19.8 ± 9.0	0.553
PLG (%)	Sysmex	80–130	92.0 ± 19.2	88.3 ± 20.0	93.3 ± 18.8	0.202
AT (%)	Sysmex	80–130	94.5 ± 15.2	90.3 ± 14.7	95.9 ± 15.1	0.069
Anti-Xa activity (IU/mL)	Werfen	–	0.05 ± 0.19	0.12 ± 0.34	0.03 ± 0.10	0.011

[a]Mean ± standard deviation

CAT, cancer-associated thrombosis; PT-INR, prothrombin time-international normalized ratio; APTT, activated partial thromboplastin time; FDP, fibrinogen/fibrin degradation products; PIC, alpha2-plasmin inhibitor-plasmin complex; FMC, fibrin monomer complex; TAT, thrombin-antithrombin complex; SF, soluble fibrin; α2-PI, alpha2-plasmin inhibitor; PAI1, plasminogen activator inhibitor-1; TM, thrombomodulin; PLG, plasminogen; AT, antithrombin

Supplementary Table 4 Prevalence of cancer-associated thrombosis (CAT) by cancer type, with venous and arterial breakdown

Cancer type	Total, n	CAT, n (%)	Venous, n	Arterial, n
Genitourinary	24	8 (33.3)	6	2
Gynecological	16	5 (31.3)	5	0
Lung	10	2 (20.0)	2	0
Gastrointestinal	65	7 (10.8)	4	3
Others	2	0 (0)	0	0
Total	117	22 (18.8)	17	5

Venous thrombosis (n = 17): deep vein thrombosis (DVT) 16, including one case with concurrent splanchnic vein thrombosis.

Arterial thrombosis (n = 5): cerebral infarction 3, peripheral arterial thrombosis 1, and abdominal aortic thrombosis 1; all clinically adjudicated as cancer-associated.

CAT, cancer-associated thrombosis
